# Medical and Philosophical Intelligence

**Published:** 1816-05

**Authors:** 


### MEDICAL AND PHILOSOPHICAL INTELLIGENCE.

*ROYAL SOCIETY.*—On Thursday, the 21st of March, a paper by SIR EVERARD HOME was read, on the mode of action of specific medicines. From experiments already made it is known that poisonous bodies, whether mineral or vegetable, do not produce their effects upon the body till they are introduced into

---

into the circulation; and the effect always follows whenever they are introduced into the circulation. Ipecacuanna injected into the jugular vein produces instant vomiting, and opium immediate drowsiness. We know at present only two specific medicines; namely, mercury for the venereal disease, and the eau medicinale, which is a vinous infusion of colchicum autumnale, for the gout. It is well known that mercury produces its effects only when introduced into the circulation. The author gives an account of several experiments with the eau medicinale on himself and on dogs, which shows that it requires likewise to be introduced into the circulation before it produces its effects.

---

*Institute of France.*—A shower of stones has fallen this year in the neighbourhood of Langres, with all the usual circumstances. M. Pistollet, physician in that town, has collected some of them. They are quite similar to other stones of the same origin, except that their fracture is perhaps a little whiter.

M. Vacquelin, who had been charged last year with the examination of the aerolites of Agen, has presented some reflections on the state in which the principal elements occur in this sort of stones. A portion of the silica appears to him to be in combination with the magnesia. Sulphur is present, united to iron; for the mineral gives sulphureted hydrogen, when dissolved in the acids. As to the chromium, it appears in a separate state, and sometimes in particles so large as to destroy all idea of combination.

---

*Identity of Galvanism and the Nervous Influence vindicated;* by Dr. Wilson Philip.—In the account in the Annals of Philosophy, of that part of a paper of mine which was read before the Royal Society on the 25th of last month, it is observed, that I go rather further than my experiments will warrant, when I conclude that the nervous influence and galvanism are the same. It is clear, it is observed, that the section of the nerve interrupts the nervous influence; and that my experiments, supposing them correct, show that galvanism puts an end to this interruption, but that it may do this merely by serving as a conductor to the nervous influence. It is impossible to receive a perfectly correct idea of all parts of a paper of this kind, from hearing it once read. From the way in which the experiment was made, there seems to be no room for the explanation here pointed out; for the cut ends of the nerve were so far from being applied, or even made to approach each other, that the upper portion was wholly neglected, and the lower portion drawn out, and coated with the tin-foil.

It has been remarked, that this experiment should have been made on some other animal than the rabbit. This suggestion comes from a quarter of such authority in physiological questions, that I have felt myself called upon to attend to it. I have repeated the experiment on dogs, and found the result, both with regard to the stomach and lungs, in all respects the same as in rabbits bits. The galvanism was not applied in such force as to occasion any expression of pain, which it does if the power of the trough is more than occasions a slight twitching in the fore legs. From these experiments, as it is observed in the paper alluded to, one of two inferences appears to be unavoidable; we must either admit the identity of the nervous influence and galvanism, or that there is a power different from the nervous influence yet capable of performing its most complicated functions, a supposition, I think, much harder to be granted than the other.

Both this experiment, and the experiments on rabbits, relating to the same subject, were made in the presence of several medical gentlemen, who expressed their entire satisfaction in the result.

*Worcester, March* 5, 1816. *Annals of Philosophy.*

---

By Mr. Field's report (in the Med. Repos.) it appears, that notwithstanding the severe, dangerous, and even fatal cases which have occurred all over the metropolis, the boys in Christ's Hospital have been unusually healthy. Were this exemption confined to mortality, we might impute it to the judicious and early treatment which the inhabitants of that establishment derive from their resident apothecary; and probably something may be attributed to that cause, as we meet with four cases of Cynanche trachealis, to the sudden arrest of which only we can impute the mildness of so many.

The cases of Cynanche parotidœa, nine in number, occurred all of them "nearly on the same day." "The infectious nature of this malady," continues the ingenious writer, "rendered it necessary to take every proper precaution to prevent its extension, which was accordingly done; and, as far as the short time now elapsed will justify the conclusion, there is great reason to believe that it will be attended with the desired effect." This attention to dates is highly important. There cannot be a doubt that this candid and acute observer will readily agree with us in the great probability that all the boys derived the disease from the same source, and not from each other. This has so often happened on-board ship, where no intercourse could be traced, that it is at least doubtful whether the disease is excited by the constitution of the influence, or by a contagious property.

---

Our readers will be anxious to learn the infantile history of Dr. Rodman's little elève, born within the fifth month of gestation.

"As several authors," observes Dr. R. "who give the size and weight of the fœtus at different periods, are inconsistent in their statements, it is necessary, in the first place, to consider the marks afforded on this subject by the catamenial discharge, since these inconsistencies must arise from some mistake.

"When the periodical appearance of this discharge returns during the influence of healthy functions, the proof that the uterus is not impregnated is satisfactory; and, when the health is good, the stoppage of this discharge is a strong proof of pregnancy.

"Along

"Along with turgescent feelings in the mammæ, and sensations of a nature peculiar to the pregnant state, the stoppage alluded to is the only mark on which mothers can rely. Hence, if a medical man be not consulted till two, three, or more months have elapsed, it is obvious that he has no other grounds by which to discover the exact term of gestation in such instances, but what he derives from the mother's history, her assertions, her appearance, and her former habits of exactness. On this account it is easy to understand how an accoucheur may be sometimes deceived, and how the tables of fœtal dimensions may often be erroneous.

"With respect to the mother of the child in question, she is tall, robust, and healthy, and was never subject to those irregular affections which derange such calculations; and, besides, her knowledge of the time of her former gestations was particularly accurate. At every new interrogation she is consistent, and modestly evasive, though her reasons are decisive; and she does not hesitate to affirm, that the period of gestation already mentioned was rather within the 19 weeks, as has been stated.

"But there are other occurrences which tend to corroborate what she affirms.

"Every accoucheur must have observed that the weight and length of children born at the full time are variable; but especially the weight. On looking into my journal of newly born children come to the full time, I find it stated that one child weighed fifteen pounds, while the weight of others was irregularly less. In whatever way it may be explained, this variation of weight is undeniable; and it is no stretch of propriety, perhaps, to grant, that according to the constitution, circumstances, and health of the mother, one fœtus at a given period differs proportionally from another fœtus at the same period.

"If it be probable that a tall, robust, and healthy female, in the vigour of life, shall have children of greater dimensions and weight, than one who is little, delicate, and unhealthy, it is equally probable that statements should contain the characteristic descriptions of the mothers before they be accounted conclusive in their proportions of the fœtus. By allowing that such statements are defective in this respect, the defects will be excusable: and the knowledge of the mother, whose opinion is the point at issue, will be admissible.

"My motive in making her son's case known, was a wish to check those unqualified assertions which are too commonly heard, that a child born at certain premature periods cannot live; such as at the sixth month, &c. And if professional men encourage this opinion, what can be expected from those that are not of the profession?

"Assertions of this nature have undoubtedly led to the death of premature infants in many instances. They seem not only to be useless, but to supersede the exertions which might save the children; and become grounds of excuse for heedless treatment, by which they are exposed to destruction. Death would have been the speedy fate of this child in the same way, had I not opposed this this assertion at his birth, and, by insisting that it was possible he might live, prompted the activity of all the necessary attentions."

"The little gentleman who has given rise to these remarks is still healthy. He became fretful three weeks ago, and would not sleep, but cried for two nights. The same cotton wool which covered his head had been too long used—new wool was put into its place and he soon got drowsy—since which he has sleeped at the usual times—his crying has ceased, and he has continued well."

---

We think it our duty, after the observations we made on Mr. FLETCHER's case (see vol. xxxiv. or No. 202, page 515), to remark, that, after a very diligent inquiry, we have met with another well-authenticated account of the re-production of a nail after the excision of part of a finger. In both, however, we are a little doubtful whether the root of the nail might not have remained. The subject whose history we last learned was very young at the time the accident happened.

---

The dreadful nature of the disease to which the subsequent article of intelligence refers, obliges us to offer it to our readers. If not altogether satisfactory, it may lead to some further attempts at subduing the heaviest affection to which the softer sex is liable.

*From the Gottingen gelehrte Anzeigen.*

"Professor OSIANDER, seven years ago, communicated to the Royal Society at Gottingen his method of cutting out the cancerous part of the uterus, (not the whole uterus, as some have erroneously imagined,) and thus to effect a cure of the cancer uteri. Though this operation, which he had performed twenty-three times, always turned out successful, yet, in many cases, a very material difficulty occurred in mollifying the indurated remainder of the uterus, and reducing the same to its natural form. A few years ago, he, however, conceived the idea of combining, in such cases, both the inward and outward application of the Aqua Laurocerasi, with the operation. This remedy operated surprisingly in different cases, after the cancerous part was cut out, in mollifying the scirrhous remains, and in the re-production of a sound substantia uteri; but he never ventured to suppose that a complete scirrhus uteri, with all the symptoms of a near approach to an open cancer, such as fever, local pains, and frequent bleedings, might be cured by the above-mentioned remedy, without any operation, till he made this experiment in the beginning of 1815, in the case of a woman of weak constitution, who, from a scrofulous cause, in and after an abortus habitualis, had got a fully indurated and thickly-swelled orificium uteri, a rough and easily-bleeding uterus, attended with spasms, a febris lenta, local pains, and frequently returning hæmorrhage. This woman, greatly afraid of the operation, requested him to try every possible means without it; to which he agreed; and began the experiment by applying the Aqua Laurocerasi only outwardly, but soon added also the inward, though very moderate use of the same, together with other requisite tonic and antispasmodic remedies, according to circumstances.

stances. This application surpassed his expectation: the swelled and indurated uterus grew softer in a short time, and by degrees regained the natural form and size. The hæmorrhage ceased entirely, and the natural order of the menses again took place. The cure was begun about the middle of Nov. 1814, and was already, in the middle of Jan. 1815, so successfully completed, that the woman, to this very day, almost a twelvemonth after, is free from this complaint, and enjoys perfect health. The efficacy of this remedy naturally consists in the very powerful operation of the prussic acid contained in the Aqua Laurocerasi, the application of which requires, however, the utmost caution."

---

Dr. Adams will commence a summer Course of Lectures on the Institutes and Practice of Medicine early in June.

Dr. Clutterbuck will begin his summer Course of Lectures on the Theory and Practice of Physic, Materia Medica, and Chemistry, early in June, at his house, No. 1, in the Crescent, New Bridge-street.

---

*Seventh Report of the General Committee of Associated Apothecaries and Surgeon-Apothecaries of England and Wales.*

*Crown and Anchor Tavern, April 24th,* 1816.

This Committee never having ceased its attention to the objects for which it was appointed, begs leave to detail its proceedings, since the last Report of January the 10th, 1815, which were published through the medium of the Medical Journals.

It is sufficiently known, that an Act, solicited by the Society of Apothecaries, passed last session, "For the better Regulating the Practice of Apothecaries throughout England and Wales."

During the passing of the Bill, many of the amendments to which former Reports of this Committee refer, were introduced; many alterations were also made in it; and, after it had passed both Houses, owing to some amendments introduced by the Peers, it was rejected by the Commons; and a new Bill, *pro forma,* was obliged to be brought in, and again be carried through Parliament. This unusual circumstance produced so much delay, that there was scarcely time to pass the second Bill through the regular stages before the close of the session.

Early in June, this Committee received a copy of a Bill "For Enlarging the Charter of the Royal College of Surgeons, &c."

The Committee approved of the principle of this Bill, generally; but objected to it—1. That it empowered the College to demand whatever sum it pleased for a diploma—2. That it continued the annual contribution levied on Members of the College residing within seven miles of London —and 3. That it contained no provisions for the regulation of the Practice of Midwifery.

The Committee, therefore, petitioned against the Bill; the effect of which was, that the College was not to exact any larger sum or sums than paid at present for diplomas, or as contributions; and that some provisions concerning Midwifery were introduced. It was the wish of the Committee, that a Board of Examiners in Midwifery should be appointed by the College. But this was positively and successfully resisted. Finally, a provision was inserted, that no male person should, *in future,* be allowed to commence practising Midwifery, for lucre or gain, except Members of the College of Surgeons, (saving the rights of the College of Physicians.) To this, as a compromise, the Committee yielded; upon the conviction, that if none hereafter were suffered to enter into this practice

 but but those who had received a medical education, and had been examined touching their knowledge in Anatomy and Surgery, there could remain no doubt that the public would be effectually guarded against the intrusion of ignorant pretenders; nor was it likely that any Member of the College would exercise the Art of Midwifery without previous instruction. The Committee of the House of Commons would not allow any mention of female midwives. This Bill meeting some obstruction in the House of Peers, and the lateness of the session preventing the last clause being obviated, it was withdrawn.

It has been introduced into Parliament in the present session; but again meeting with objections, the College did not persist and press for a division on it.

There is no official authority for mentioning it, but the Committee, from private information, have some reason for believing that another Bill will be arranged, founded upon more enlarged and disinterested views.

This Committee did not expect that an Act so novel in its principles as the Apothecaries, would be free from errors; indeed many of its defects had been pointed out in the debate which took place upon it in the House of Peers. Some of these unquestionably resulted from the confusion attendant on the untoward circumstances with which the Bill was passed, and the time before the session would close did not admit of remedying them. During the recess, the Committee saw with extreme pain, that there were more errors in the Act than were at first observed; and that its operation was in some parts retrospective, and consequently unjust.

As the Act had been taken out of the hands of this Committee, and was solicited by the Society of Apothecaries, the former entertained no doubt that the latter would bring in a Bill this session, to amend whatever errors existed in it. The Committee waited the meeting of Parliament; and, finding time passing on without any movement of the Society for the purpose, conceived it an indispensable duty to apply to that body for an explanation of its intentions.

The following resolutions were therefore passed and transmitted to the Society, with the request of an early answer.

(COPY.)

GENTLEMEN, 62, *Gower-street, March 23d*, 1816.

Pursuant to a resolution of the General Committee of Apothecaries and Surgeon-Apothecaries of England and Wales, I have the honour to present to you a copy of the Resolutions passed at a Meeting held on the 19th inst. to which the Committee request an early answer.

I have the honour to be, Gentlemen, your obedient humble servant,

G. M. BURROWS.

*To the Master, Wardens, and Court of Assistants, of the Society of Apothecaries.*

At a Meeting of the General Committee, held on March 19th ult. it was

Resolved,

"That, as the provisions (see sections 14 and 15) of the Act "for the better Regulation of the Practice of Apothecaries" have a retrospective effect, in consequence of certain unexpected alterations being made in the Bill, which effect was not contemplated by the framers of that Bill, and which has been found extremely prejudicial to the interests of numerous Medical Students and others;

Resolved,

"Therefore, that a respectful appeal be made to the Society of Apothecaries, to apply to Parliament during the present Session, for the purpose of making such amendments in the Act as shall obviate its retrospective operation.

To

To which this communication was returned:

SIR, *Apothecaries' Hall, 9th April,* 1816.

I am directed by the Committee appointed by the Master, Wardens, and Court of Assistants, to whom your letter of the 19th of March, together with two Resolutions of your General Committee, were referred, to acknowledge the receipt thereof; and to inform you that the same will be taken into consideration at the next meeting of the Committee.—I am, Sir, your obedient humble servant, J. BACKLER, Clerk.

*To G. M. Burrows, Esq.*

This proving very unsatisfactory, the Chairman was requested to apply for a definite answer; when he again wrote—

GENTLEMEN, 62, *Gower-street, April 17th,* 1816.

At a Meeting held this evening of the General Committee of Apothecaries and Surgeon-Apothecaries of England and Wales, I was requested to communicate to you, that the Meeting was adjourned to this day week, for the purpose of *then* receiving and taking into consideration an answer to the Resolutions of the Committee of the 19th ult., and which I had the honour of transmitting.

And I am further instructed to request that the said answer be a definite one to the subject of those Resolutions.

I have the honour to be, Gentlemen, your obedient humble servant,

G. M. BURROWS, Chairman.

*To the Master, Wardens, and Society of Apothecaries.*

On the 19th, the following was received:

SIR, *Apothecaries' Hall,* 19 *April,* 1816.

I am directed by the Bill Committee of the Court of Assistants to transmit to you a copy of their Resolution of the 16th instant.—I am, Sir, your obedient humble servant, J. BACKLER, Clerk to the Society.

*To Dr. G. M. Burrows.*

At a Meeting of the Bill Committee appointed by the Court of Assistants held at Apothecaries' Hall, on Tuesday the 16th of April, 1816.

The Committee having taken into consideration the Letter addressed to the Court by Dr. G. M. Burrows, with the Resolution of the General Committee of Apothecaries and Surgeon-Apothecaries of England and Wales, calling upon them to apply to Parliament for an amended Act,

Resolved,

"That it does not appear to this Committee that any such practical inconvenience has arisen from the alledged defects of the Act of last Session as to induce this Committee to recommend to the Court of Assistants any immediate application to Parliament."

As it was well ascertained by your Committee, that the Society of Apothecaries was fully apprised of the *practical inconveniencies that had arisen from the alledged defects* in the Act, it was clear, from this Resolution, it was determined not to apply to Parliament, this session at least, to amend it.

This decision the Committee exceedingly laments. Deeply impressed with the greatness of the injury sustained by several classes of the medical profession, from the errors in the Act, and hence its inadequateness for the objects intended; and, sincerely deploring the consequences, it feels bound to adopt every means in its power to seek that redress the extent and urgency of the evil demand. But, previously to any other steps being taken, this Committee requires more specific information than it yet possesses, of the injuries and inconveniencies experienced from the operation of the Act.

Much information has been received by various Members of the Committee on the subject of the Act, but not in that capacity, but as private individuals. None of the District Committees have communicated with the the General Committee since the publication of the Sixth Report of January, 1815; nor have any individuals addressed it.

This Committee is consequently without official data on which to found any further proceedings, that may lead to the attainment of that object which it avowedly professes—the amendment of the Apothecaries' Act.

The Resolution annexed,* which has been advertised very generally, it is hoped will procure the necessary documents, and evince to the profession, that this Committee is ever alive to the interests of its constituents, and will never abandon a trust so honourably bestowed, and with so much confidence continued. G. M. Burrows, Chairman.

---

*To the Editors of the London Medical and Physical Journal.*

Gentlemen,

I did myself the pleasure of stating to you, last year, my intention of reviving the mode of teaching botany, as used to be practised by my late friend and partner Mr. William Curtis; and having made an experiment, and finding it likely to answer my own purpose, and that of the gentlemen who honoured me with their attendance, I have been induced this season to commence the same again; and, as it may be useful to some of your readers to know at what season the different plants are to be found in bloom in the neighbourhood of London, I propose to give you for insertion every month a list of such as we meet with, and their places of growth. The present spring is uncommonly backward, a circumstance which will be worth recording for future comparison. As I propose giving you the same kind of list for two years, it will be a subject of curiosity to observe the contrast: nothing will point out the difference of seasons more than the period of plants blooming, and at the same time afford you a complete calendar of flora for future reference.

I met the gentlemen comprising my class on Tuesday the 23d instant, at the Red House, Battersea Fields; and the following scanty list of plants was all that we met with, and consequently all that was in bloom at that season.

*Tussilago Petasites*—Butter burr.

———— *farfara*—Coltsfoot.

*Glechoma hederacea*—Ground ivy.

*Veronica hederæfolia* — Ivy-leaved speedwell.

———— *agrestis*—Small blue ditto.

*Caltha palustris*—Marsh marigold.

*Draba verna*—Whitlow grass.

*Bellis perennis*—Daisy.

*Chærophyllum sylvestre* (but just out)—Cow parsley.

*Cardamine pratense* (scarcely a petal open)—Ladies' smock.

*Ranunculus Ficaria*—Pile-wort.

*Senecio vulgaris*—Grounsel.

*Thlaspi Bursa Pastoris*—Shepherd's purse.

*Salix fragilis*—Crack willow.

——— *alba*—White ditto.

——— *vitellina*—Golden ditto.

——— *viminalis*—Ozier.

*Betula alba* (scarcely out) White birch.

Our next excursion will be on Friday the 8th instant, at the Red House, Battersea, at ten o'clock in the morning; on Friday the 19th, at the Spaniard, Hampstead Heath; on Tuesday the 23d, at the Robin Hood, at Kingston Bottom; and on Friday following, at the French Horn, Charlton,—all at the same hour.

I am, Sir, your obedient Servant,

W. Salisbury.

---

* See our present Wrapper.

### METEOROLOGICAL REGISTER.

*From March the 26th, to April the 25th,* 1816.

Kept by C. BLUNT, Philosophical Instrument Maker, No. 38, Tavistock-Street, Covent-Garden.

| Moon. | Day. | Wind. | Barometrical Pressure. Max. | Min. | Mean. | Temperature. Max. | Min. | Mean. | |
|---|---|---|---|---|---|---|---|---|---|
| | 26 | SE | 30·21 | 30·18 | 30·195 | 48 | 35 | 41·5 | Fair |
| | 27 | E | 30·25 | 30·23 | 30· 24 | 50 | 36 | 42· | Fair |
| ● | 28 | E | 30·27 | 30·25 | 30· 26 | 49 | 34 | 41·5 | Fair |
| | 29 | E | 30·29 | 30 28 | 30·285 | 49 | 33 | 41· | Fair |
| | 30 | NE | 30·27 | 30·20 | 30·235 | 49 | 33 | 41· | Fair |
| | 31 | N | 30·10 | 29·80 | 29· 95 | 50 | 26 | 38· | Fair |
| | 1 | NW | 30· | 29 86 | 29· 93 | 49 | 29 | 39· | Fair |
| | 2 | NW | 30·16 | 30· | 30· 08 | 50 | 31 | 40 5 | Fair |
| | 3 | NW | 30·37 | 30·24 | 30·305 | 49 | 32 | 40·5 | Fair |
| | 4 | W | 30 42 | 30·25 | 30·335 | 48 | 28 | 38· | Fair |
| ◐ | 5 | W | 30 24 | 30·16 | 30· 20 | 50 | 32 | 41· | Fair |
| | 6 | NE | 30 20 | 30·10 | 30· 15 | 50 | 33 | 41 5 | Fair |
| | 7 | NE | 30·10 | 30· | 30· 05 | 49 | 33 | 41· | Fair |
| | 8 | NE | 29·90 | 29 85 | 29·825 | 48 | 32 | 40· | Fair |
| | 9 | E | 29·87 | 29·86 | 29·865 | 49 | 30 | 39·5 | Fair |
| | 10 | SE | 29·90 | 29 88 | 29· 89 | 52 | 36 | 44· | Fair |
| ○ | 11 | E | 29·56 | 29 55 | 29·555 | 51 | 35 | 43· | Fair |
| | 12 | E | 29·58 | 29·56 | 29· 57 | 50 | 35 | 42·5 | Snow |
| | 13 | NE | 29·61 | 29·59 | 29· 60 | 52 | 33 | 42·5 | Snow |
| | 14 | E | 29·60 | 29·56 | 29· 58 | 50 | 28 | 39· | Snow |
| | 15 | NE | 29·54 | 29·52 | 29· 53 | 50 | 29 | 39·5 | Hail |
| | 16 | SW | 29·53 | 29·51 | 29· 52 | 52 | 32 | 42· | Rain |
| | 17 | S | 29·60 | 29·50 | 29· 55 | 53 | 38 | 44·5 | Fair |
| | 18 | S | 29·79 | 29·70 | 29·745 | 53 | 33 | 43· | Fair |
| ◑ | 19 | SE | 29 82 | 29·81 | 29 815 | 54 | 40 | 47· | Fair |
| | 20 | E | 29·80 | 29·70 | 29· 75 | 52 | 35 | 43·5 | Rain |
| | 21 | E | 29·74 | 29·72 | 29· 73 | 54 | 38 | 46· | Rain |
| | 22 | SE | 29·78 | 29·76 | 29· 77 | 56 | 45 | 50·5 | Rain |
| | 23 | SE | 30· | 29·80 | 29· 90 | 58 | 48 | 53· | Rain |
| | 24 | SE | 30· | 30· | 30· 00 | 63 | 48 | 55·5 | Fair |
| | 25 | SE | 30·09 | 30·06 | 30·075 | 64 | 49 | 56·5 | Fair |

RESULTS.

Mean barometrical pressure of the month 29·886
Maximum 30·42, wind at W
Minimum 29·50, ——— S

Mean temperature of the month 43·193 deg.
Maximum 64, wind at SE
Minimum 26, ——— N

*Scale exhibiting the prevailing Winds during the Month.*

| N | NE | E | SE | S | SW | W | NW |
|---|---|---|---|---|---|---|---|
| 1 | 6 | 9 | 7 | 2 | 1 | 2 | 3 |

| | Mean barometrical pressure. | Mean temperature. |
|---|---|---|
| From the new moon on the 28th March, to the first quarter, on the 5th April | 30·172 | 39·937 |
| ——— the first quarter on the 5th, to the full moon on the 11th | 29·996 | 41·16 |
| ——— the full moon on the 11th, to last quarter on the 19th | 29·581 | 42·125 |

REPORT

### REPORT OF DISEASES.

The same disposition to high inflammation marks all the diseases of the last month, whether ophthalmia, rheumatism, gastritis, or pleuritis, or in whatever part the pain has been intense; the pulse for the most part hard, and the blood taken buffy and cupped. Inveterate uterine pains have been greatly exasperated, and much relieved by the lancet. In one case, of four years standing, the blood drawn, at the second bleeding of two successive days, to the amount of twelve ounces, exhibited all the marks of high inflammation. The patient was bled the last time *ad deliquium*, and with great advantage; but it seems probable that the operation must be repeated, though not to the same extent. The reporter is persuaded that by a proper attention to successive or periodical bleedings, many of those dreadful cases which end in the malignant ulcer of the uterus might be prevented.

In one case, apparently of pericarditis, after the third bleeding on the third day of the acute symptoms, lipothymia with convulsions followed. The patient, however, mended, but did not become free from threatening symptoms without several local bleedings and other evacuations.

The method found the most successful has been early free bleedings *ad deliquium*, after which a frequent recourse to the lancet or cupping as soon as any pain or symptoms of inflammation returned. In these bleedings the quantity taken was limited by the effect produced.

Cutaneous diseases are increasing as the season advances, and as the poorer classes resort to the metropolis preparatory to their being engaged in agricultural employments, or, to use their own language, as "the sun gets on both sides the hedge." Towards the close of the month colicky and bilious attacks have been frequent, but not dangerous.

Colchicum still retains its reputation in gout. We shall, in a future Number, give an historical account of the changes in the preparation of this plant, till at last a combination has been discovered which may be given with safety, and, for the most part, with success.

