# Errata

**Published:** 1816-06

**Authors:** 


—— 424, —— 12 ———, for *influence* read *atmosphere.*

